# Supplementary material for: Origin of the multi-phasic quenching dynamics in the BLUF domains across the species
Source: Nat Commun. 2024 Jan 20;15:623. doi: 10.1038/s41467-023-44565-5 (PMC10799861; doi:10.1038/s41467-023-44565-5)
Supplement: Supplementary file 1 — Supplementary Information [file 41467_2023_44565_MOESM1_ESM.pdf]

Supplementary Information

Origin of the Multi-phasic Quenching Dynamics in the BLUF Domains Across the Species

Yalin Zhou[+1], Siwei Tang[+1], Zijing Chen[1], Zhongneng Zhou[1], Jiulong Huang[1], Xiu-Wen Kang[1], Shuhua Zou[1], Bingyao Wang[1], Tianyi Zhang[1], Bei Ding[1*], and Dongping Zhong[1,2*]

[1]Center for Ultrafast Science and Technology, School of Chemistry and Chemical Engineering, Shanghai Jiao Tong University, Shanghai 200240, China

[2]Department of Physics, Department of Chemistry and Biochemistry, and Programs of Biophysics, Chemical Physics, and Biochemistry, The Ohio State University, Columbus, Ohio 43210, United States

[*]Correspondence author: Prof. Bei Ding (E-mail: bei.ding@sjtu.edu.cn), Prof. Dongping Zhong (E-mail: zhong.28@osu.edu)

[+]Co-first authors.

Key words: Structural heterogeneity, proton-coupled electron transfer, non-natural amino acids, transient absorption, blue-light photoreceptors, ^{19}F NMR probe

Supplementary Figures

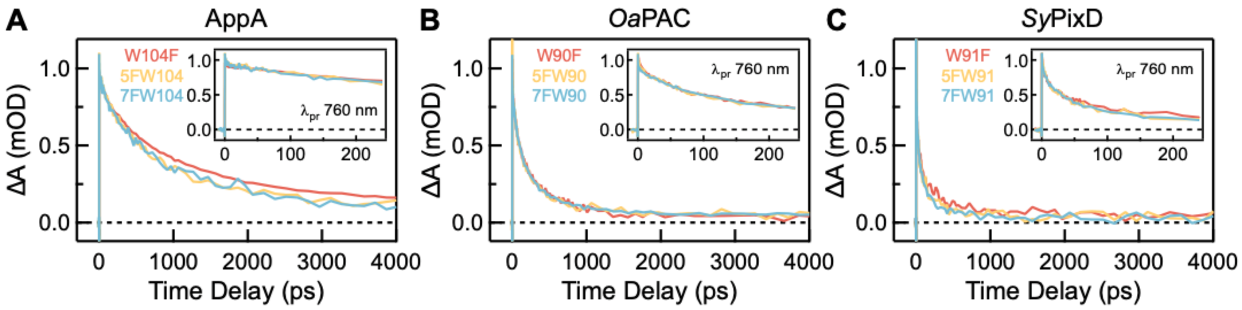

Supplementary Fig. 1 | Overlapping kinetics at 760 nm of WnF and FW labeled WT protein. The dynamics of WnF (red lines), 5FW labeled WT (orange lines) and 7FW labeled WT (blue lines) in AppA (**A**), *Oa*PAC (**B**) and *Sy*PixD (**C**) are displayed up to 4000 ps. Insets show the initial dynamics up to 250 ps.

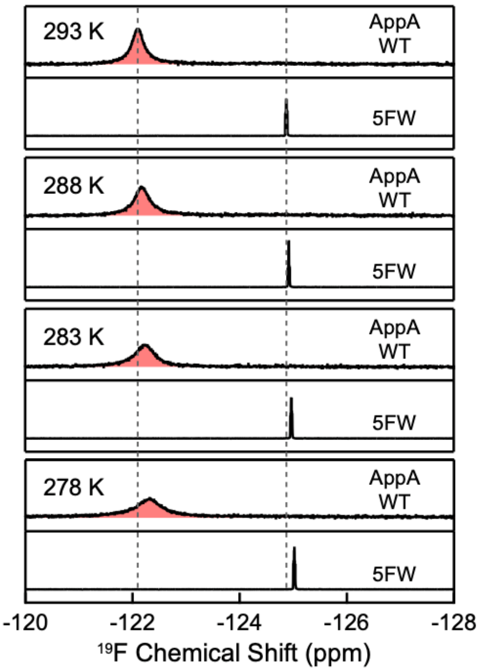

Supplementary Fig. 2 | The ^{19}F spectra for 5FW labeled AppA WT and free 5FW at 278 K, 283 K, 288 K and 293K.

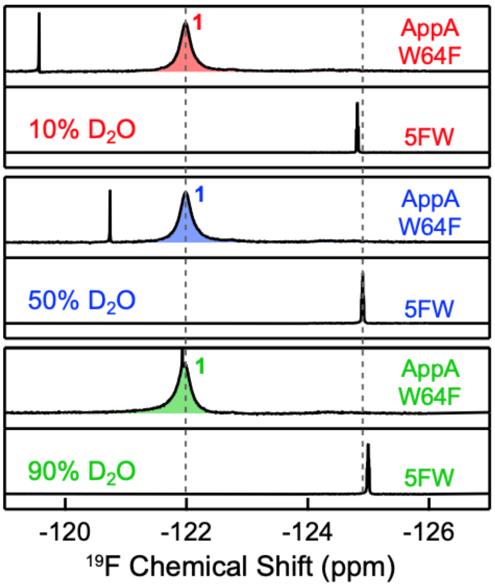

Supplementary Fig. 3 | The ^{19}F spectra for 5FW labeled AppA WT and free 5FW with increasing D_2O ratio. The single Peak 1 of the AppA WT remains unchanged with 10% (red), 50% (blue), and 90% (green) D_2O/H_2O, whereas the peaks for the free 5FW molecule and an impurity (resonance at -119.6 ppm[1] in 10% D_2O) shift upon increasing the D_2O ratio. The chemical shifts are listed in Supplementary Table 2.

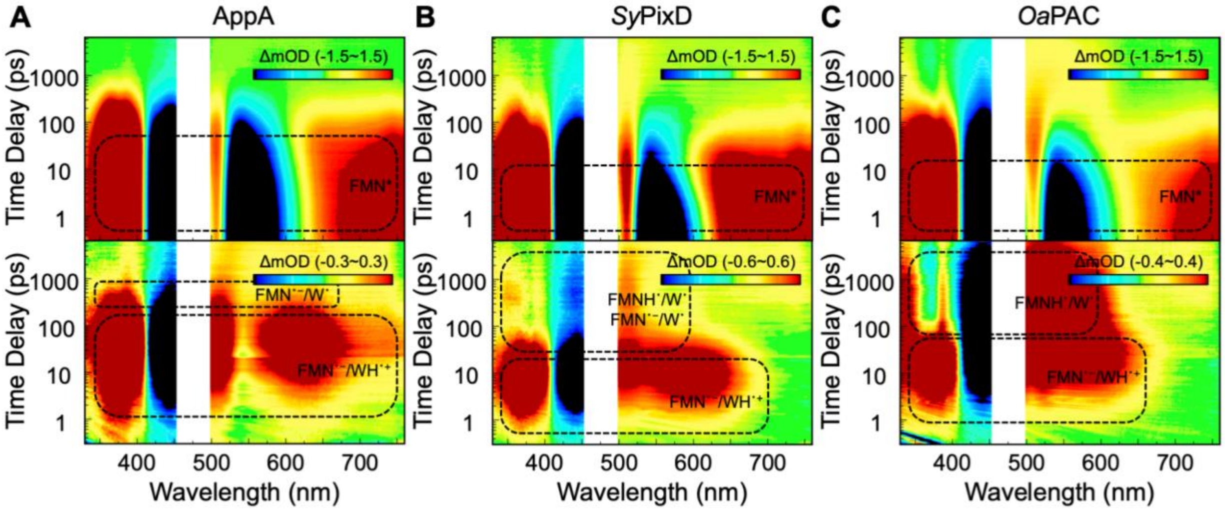

Supplementary Fig. 4 | Transient absorption 2D contour maps for YnF mutants in D_2O buffer. For AppA Y21F (**A**), *Sy*PixD Y8F (**B**), and *Oa*PAC Y6F (**C**), the upper panel shows the original TA 2D spectra, and the lower panel shows the 2D spectra after subtracting the contribution from FMN* component. Detected transient species are denoted with dashed circles with internal annotations representing possible intermediates.

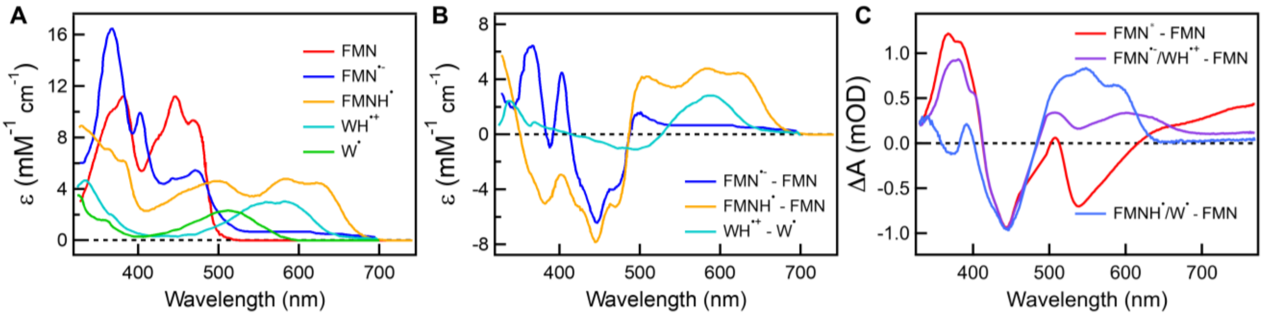

Supplementary Fig. 5 | Reference spectra. (A) Reference spectra of FMN (red), FMN$^{\cdot-}$ (blue), FMNH$^{\cdot}$ (orange), WH$^{\cdot+}$ (cyan), and W$^{\cdot}$ (green).[2] (**B**) The difference spectra between FMN$^{\cdot-}$ and FMN (blue), FMNH$^{\cdot}$ and FMN (orange), WH$^{\cdot+}$ and W$^{\cdot}$ (cyan), according to the reference spectra taken from (**A**). (**C**) The difference spectra between FMN* and FMN (red), FMN$^{\cdot-}$/WH$^{\cdot+}$ and FMN (purple), FMNH$^{\cdot}$/W$^{\cdot}$ and FMN (blue) in *Oa*PAC FMN-Gln-Trp motif.[3]

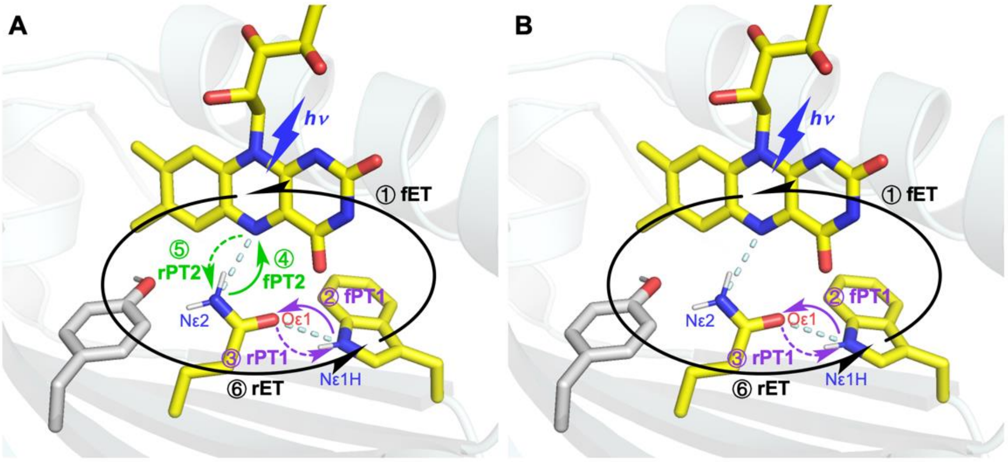

Supplementary Fig. 6 | The proposed mechanism schemes for the YnF photochemistry in three BLUF domains. The photo-reaction proceeds with six elementary steps, where the arrows represent the direction of the electron (half arrow) and proton flow (full arrow). Step 1 and step 6 are the forward and reverse ET after light-excitation (black half arrows). Step 2 and step 3 are the forward and reverse PT1 (purple full arrows), representing the first proton rocking process. Step 4 and step 5 are the forward and reverse PT2 (green full arrows), representing the second proton rocking process. *Sy*PixD and *Oa*PAC undergo scheme (**A**), while AppA follows scheme (**B**).

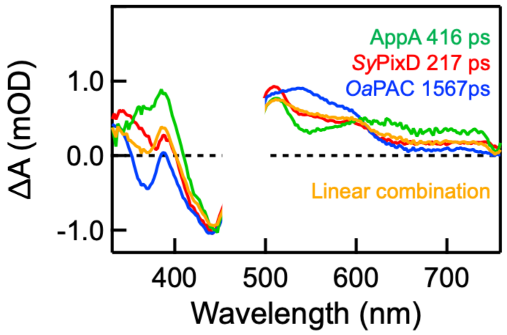

Supplementary Fig. 7 | Spectrum decomposition of *Sy*PixD Y8F spectrum. *Sy*PixD 217 ps spectrum (red line) can be decomposed into a linear combination of the green line (AppA 416 ps spectrum, PR state) and the blue line (*Oa*PAC 1567 ps spectrum, DR state). The linear combination is represented by the orange line, calculated as the sum of the green line multiplied by 0.5491, the blue line multiplied by 0.4493. The similarity in the lineshape of the red line to the orange line suggests that the 217 ps spectrum for *Sy*PixD contains a mixture of PR and DR state. The subtle difference can be attributed to the discrepancy in their steady-state UV/Vis absorption, especially in the UV region, as shown in Supplementary Fig. 17.

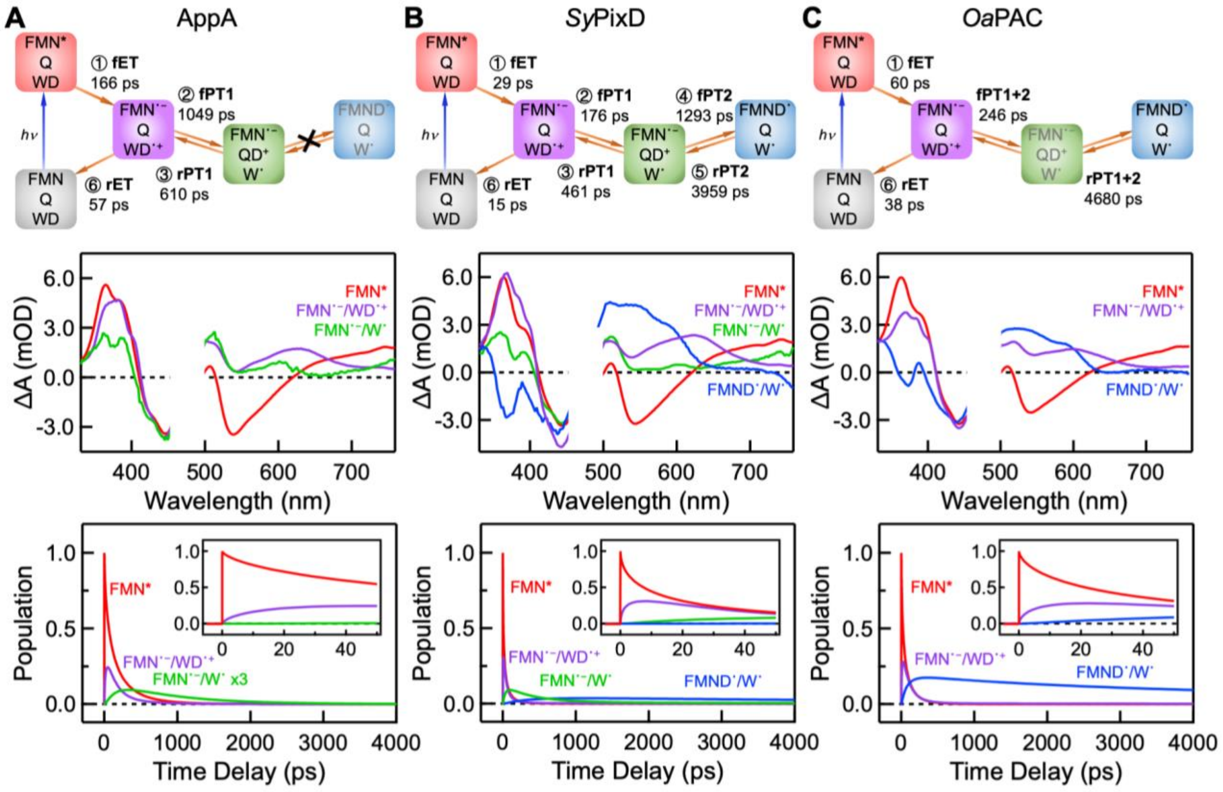

Supplementary Fig. 8 | Target analysis results of YnF mutants in D_2O buffer. A-C columns show the target analysis results of AppA Y21F (**A**), *Sy*PixD Y8F (**B**), and *Oa*PAC Y6F (**C**) in D_2O condition, respectively. The

upper panels illustrate the proposed kinetic models, single rocking (**A**) and double rocking models (**B**, **C**), for the photoreaction cycle. The arrows depict pathways with fitted averaged lifetimes, while the symbol "×" in (**A**) indicates that PT2 is inaccessible within our time window for the AppA Y21F mutant. In the middle and lower panels, the species-associated differential spectra (SADS) and corresponding kinetic traces are presented, FMN^* is depicted in red, $FMN^{\cdot-}/WH^{\cdot+}$ in purple, $FMN^{\cdot-}/W^{\cdot}$ in green, and $FMNH^{\cdot}/W^{\cdot}$ in blue. The inserts are the initial dynamics up to 50 ps.

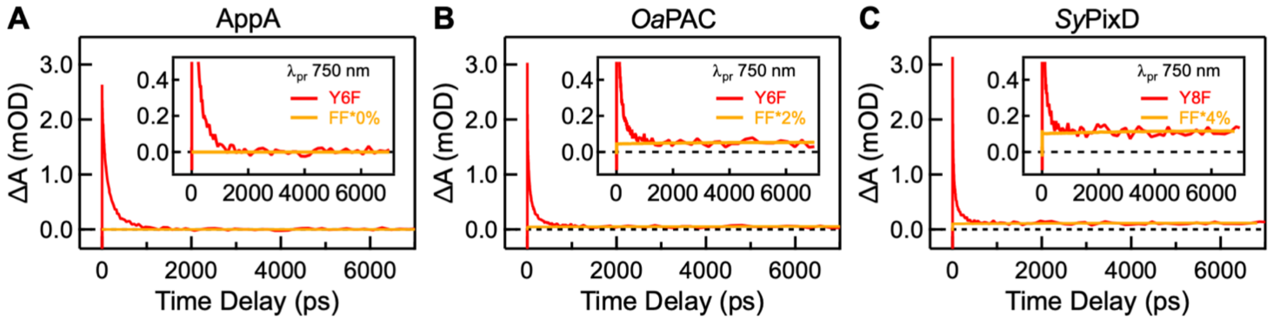

Supplementary Fig. 9 | The percentages of W_{out} configuration determined by TA spectroscopy. As shown in Supplementary Table 3 and Supplementary Table 4, when the central Tyr and nearby Trp residues were mutated into ET-inert Phe (referred to as YnF/WnF mutant), the quench rate of FMN^* was significantly slowed down compared to YnF mutants in all three species. The only pathway observed in the double FF mutants is the internal system crossing (ISC), leading to the formation of ^3FMN. The contribution of ^3FMN from the $W_{in}NH_{in}$ configuration is negligible due to the efficient electron transfer (ET). In this figure, the quenching of FMN* (red lines) is significant among the three species, a minor yet noticeable 750 nm offset (orange lines) is observed in *Oa*PAC (**B**) and *Sy*PixD (**C**), indicating the presence of the W_{out} configuration, while AppA (**A**) shows no existence of W_{out}, which is consistent with the 5FW and 7FW ^{19}F NMR results (Supplementary Table 1).

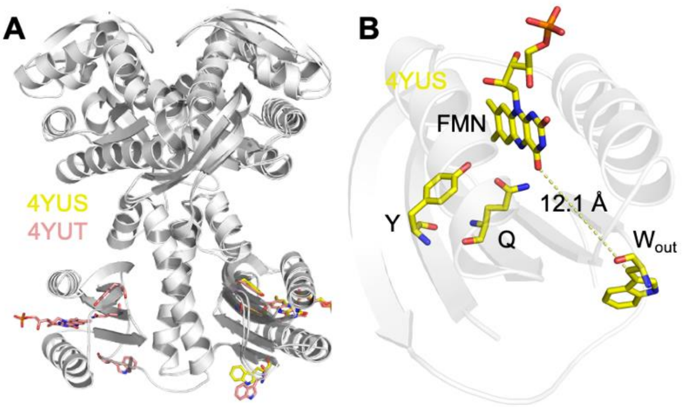

Supplementary Fig. 10 | X-ray structures of dark-state full-length *Oa*PAC and its active site. (**A**) X-ray structure of dark-state full-length *Oa*PAC$_{1\text{-}366}$ (PDB: 4YUS and 4YUT). The central FMN, Gln, Tyr and Trp are presented as sticks in yellow (4YUS) or salmon (4YUT), while other secondary structures are displayed in grey cartoon. (**B**) The structure of BLUF domain *Oa*PAC$_{1\text{-}102}$ and the active site (PDB: 4YUS). The Trp residue in three subunits all adopt the W$_{out}$ conformation. In this configuration, the distance (represented by a dashed line in **B**) between Trp and FMN is >10 Å, making efficient ET challenging.

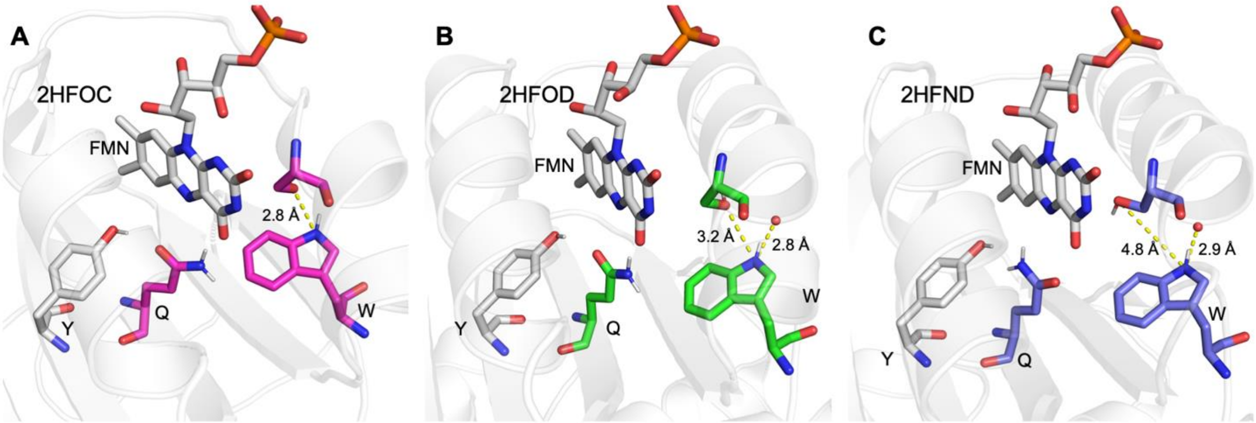

Supplementary Fig. 11 | X-ray structures of the active site of dark-state *Sy*PixD. *Sy*PixD X-ray structures (PDB: 2HFOC, 2HFOD, 2HFND) revealing W$_{in}$NH$_{out}$ subpopulations, with the Gln, Trp and Ser colored in 2HFOC (pink in **A**), 2HFOD (green in **B**) and 2HFND (slate in **C**). The structures demonstrate that when three subunits adopt the W$_{in}$NH$_{out}$ conformation, their local environments exhibit variations. The Peak 2 and 2' resonances in both 5FW and 7FW ^{19}F NMR spectra likely originate from the structural heterogeneity around Trp.

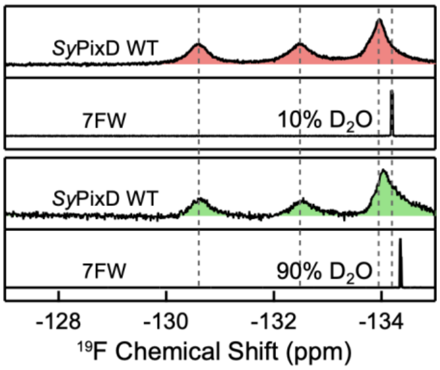

Supplementary Fig. 12 | The ^{19}F spectra for 7FW labeled *Sy*PixD WT and free 7FW with increasing D_2O ratio. The peaks in 10% D_2O and 90% D_2O conditions are shown in red and green, respectively.

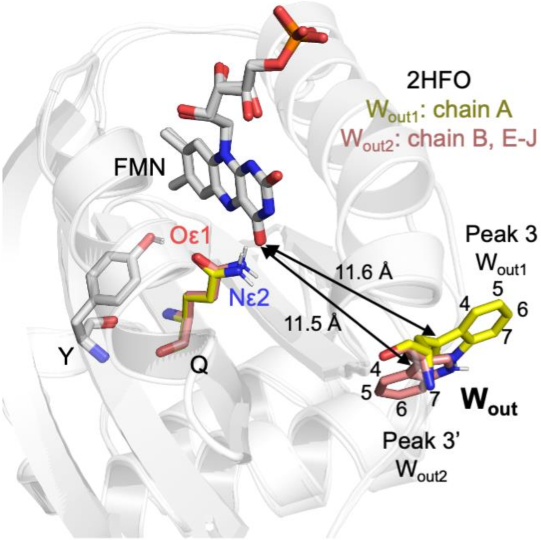

Supplementary Fig. 13 | *Sy*PixD X-ray structures (PDB: 2HFO) revealing W_{out} subpopulations. The X-ray structure of *Sy*PixD (PDB: 2HFO) provides insights into the presence of subpopulations corresponding to Peak 3 and 3' observed in both the 5FW and 7FW ^{19}F NMR spectra. The 2HFO structure consists of ten subunits, where chains C and D adopt the $W_{in}NH_{out}$ conformation and the remaining eight subunits adopt the W_{out} conformation. Among these eight W_{out} subunits, only one subunit (chain A) exhibits the W_{out1} configuration and the other seven subunits contain W_{out2} configuration.

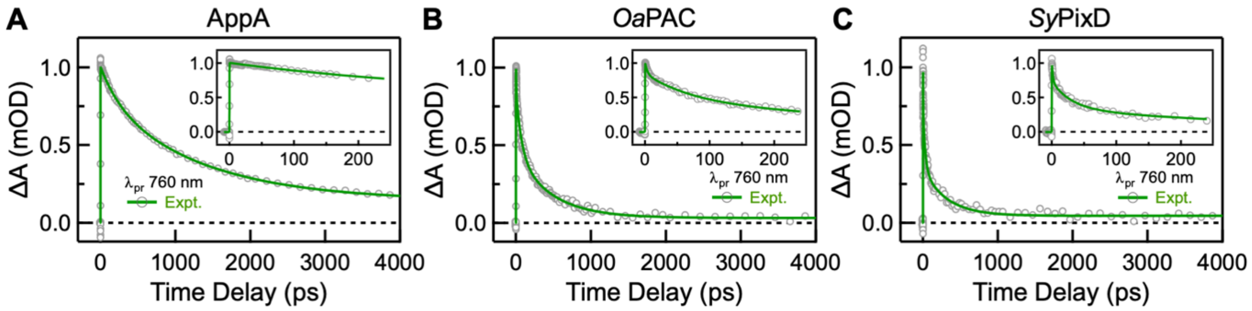

Supplementary Fig. 14 | Kinetic transients at a selective probe wavelength of 760 nm. A multi-exponential function was used to fit the kinetic traces of AppA W104F (**A**), *Oa*PAC W90F (**B**), and *Sy*PixD W91F (**C**). The raw data is depicted as symbols (○), while the fitted traces are represented by solid green lines. Insets show the initial dynamics up to 250 ps. The parameters used in the fitting are listed in Supplementary Table 4.

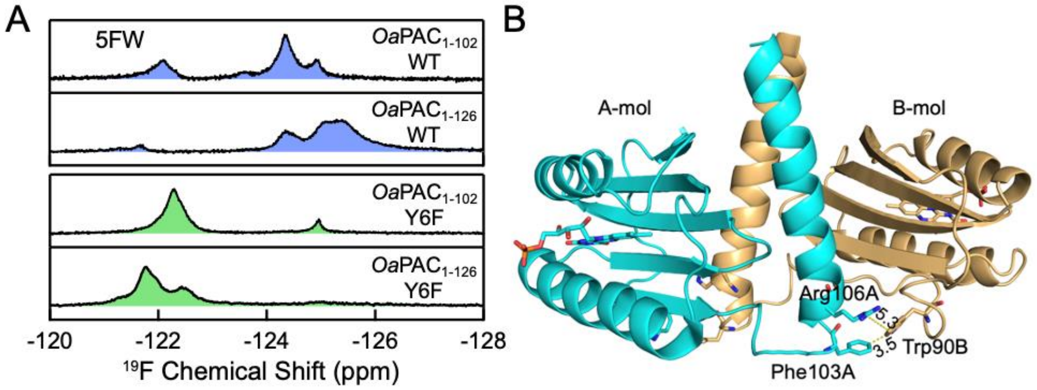

Supplementary Fig. 15 | The ^{19}F NMR spectra for 5FW labeled *Oa*PAC$_{1\text{-}102}$ and *Oa*PAC$_{1\text{-}126}$. (**A**)The ^{19}F NMR spectra for 5FW labeled WT of *Oa*PAC$_{1\text{-}102}$ (blue) and *Oa*PAC$_{1\text{-}126}$ (green) and corresponding Y6F mutants. (**B**) The W$_{out}$ configuration of *Oa*PAC$_{1\text{-}366}$ contacts Phe103 of the partner chain (PDB code: 4YUT), with A-mol in cyan and B-mol in brown.

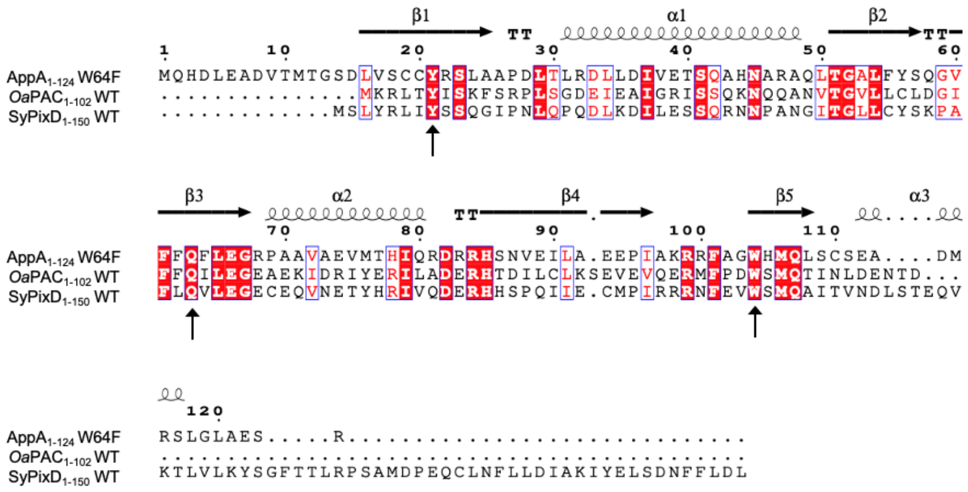

Supplementary Fig. 16 | The sequence alignment figure of the WT protein. The critical positions of the central Tyr, Gln and Trp mentioned in the main text are labeled with black arrows.

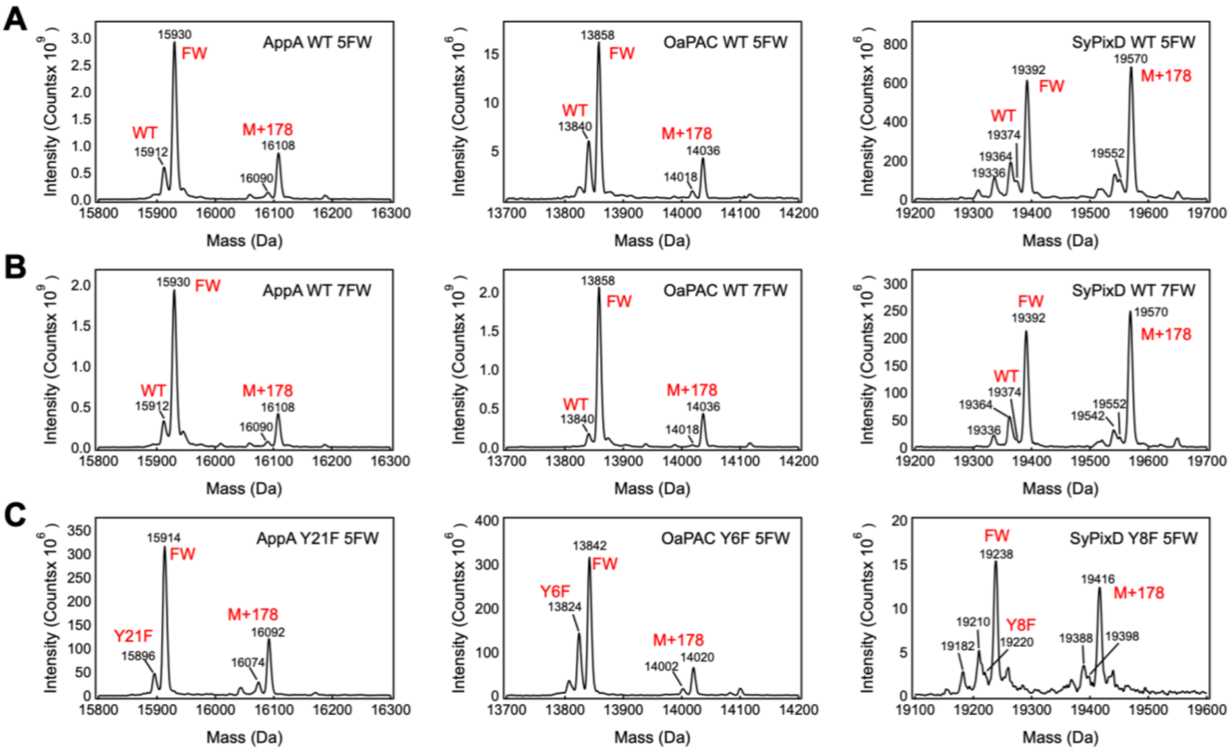

Supplementary Fig. 17 | The incorporation levels of tryptophan analogues as determined by liquid chromatography-mass spectrometry (LC-MS) analysis. The upper panel (**A**) shows the analysis results of 5FW-incorporated WT samples, the middle panel (**B**) shows that of 7FW-incorporated WT samples and the lower panel (**C**) shows 5FW-incorporated YnF mutant samples. The masses of both the WT and fluorotryptophan-incorporated proteins are provided, and the incorporation efficiency ranges from 70% to 98%. Additionally, a mass shift of M+178 was observed corresponding to the posttranslational modifications present in all the proteins.[4]

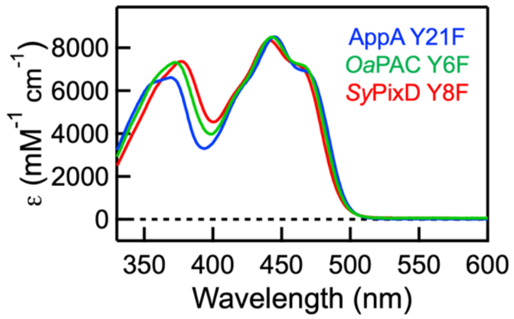

Supplementary Fig. 18 | Normalized steady-state UV/Vis absorption spectra of three YnF mutants, AppA Y21F (blue), *Oa*PAC Y6F (green), *Sy*PixD Y8F (red)**.**

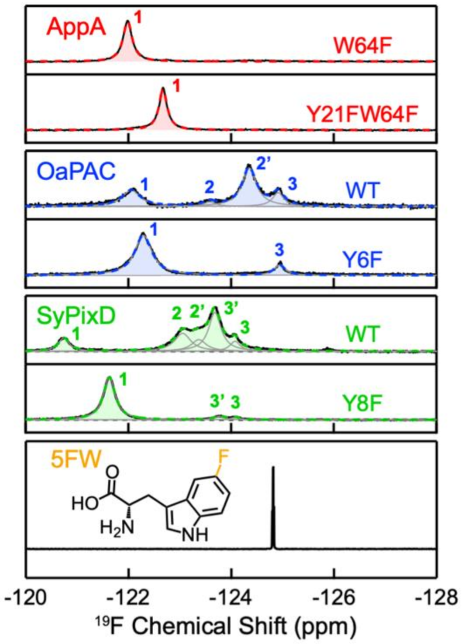

Supplementary Fig. 19 | ^{19}F solution NMR results of 5FW labeled WT and YnF mutants of three BLUF domains and free 5FW in 10% D_2O at 298 K. The fitted ^{19}F NMR spectra of AppA, *Oa*PAC, and *Sy*PixD BLUF domains are displayed as dashed red, blue, and green, respectively, while the rawdata are presented as solid black curves. Their resolved peaks are labeled (1, 2/2', 3/3'). Deconvolution of the 5FW ^{19}F NMR spectra (Fig. 2) into several Lorentzians (solid grey lines) reveals the estimated chemical shifts, line widths and the percentages of the areas under the peaks, available in Supplementary Table 1. The chemical structure of 5FW, with fluorine atom labeled in orange, is depicted in the lower panel.

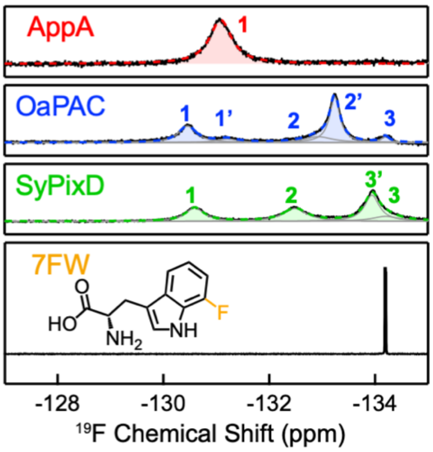

Supplementary Fig. 20 | ^{19}F solution NMR results of 5FW labeled WT of three BLUF domains and 5FW alone in 10% D_2O at 298 K. The fitted ^{19}F NMR spectra of AppA, *Oa*PAC, and *Sy*PixD BLUF domains are displayed as dashed red, blue, and green, respectively, while the rawdata are presented as solid black curves. Their resolved peaks are labeled (1/1', 2/2', 3/3'). Deconvolution of the 7FW ^{19}F NMR spectra (Fig. 5) into several Lorentzians (solid grey lines), the estimated chemical shifts, line widths and the percentages of the areas under the peaks can be found in Supplementary Table 1. The chemical structure of 7FW, with fluorine atom labeled in orange, is depicted in the lower panel.

Supplementary Table

Supplementary Table 1. Spectrum deconvolution as the sum of several Lorentzian peaks (Supplementary Fig. 19 for Fig. 2 and Supplementary Fig. 20 for Fig. 5) using $L\,(\omega, \omega_0, \gamma) = \frac{2}{\pi\gamma}\frac{1}{\left[1+4(\frac{\omega-\omega_0}{\gamma})^2\right]}$, ω_0 is the location of the peak of the distribution, and γ serves as a scaling parameter specifying the FWHM. The estimated chemical shifts ω_0 and line widths γ are given in ppm. The areas under the peaks are provided as percentages (%). The chemical shift of free 5FW and 7FW are -124.8 and -134.2 ppm, respectively.

	^{19}F	1	1’	2	2’	3	3’
		$W_{in}NH_{in}$		$W_{in}NH_{out}$		W_{out}	
AppA Y21F 5FW	Chemical shift (ppm)	-122.7					
	Line width (ppm)	0.20					
	Intergral (%)	100					
	Total (%)	100					
AppA WT 5FW	Chemical shift (ppm)	-122.0					
	Line width (ppm)	0.20					
	Intergral (%)	100					
	Total (%)	100					
AppA WT 7FW	Chemical shift (ppm)	-131.1					
	Line width (ppm)	0.51					
	Intergral (%)	100					
	Total (%)	100					
*Oa*PAC Y6F 5FW	Chemical shift (ppm)	-122.3				-124.9	
	Line width (ppm)	0.41				0.26	
	Intergral (%)	86				14	
	Total (%)	86				14	
*Oa*PAC WT 5FW	Chemical shift (ppm)	-122.1		-123.6	-124.4	-124.9	
	Line width (ppm)	0.43		0.29	0.34	0.26	
	Intergral (%)	28		5	52	15	
	Total (%)	28		57		15	
*Oa*PAC$_{126}$ Y6F 5FW	Chemical shift (ppm)	-121.8	-122.5			-125.0	
	Line width (ppm)	0.46	0.56			0.80	
	Intergral (%)	60	28			12	
	Total (%)	88			12		
*Oa*PAC$_{126}$ WT 5FW	Chemical shift (ppm)	-121.6		-124.4		-125.0	-125.4
	Line width (ppm)	0.39		0.30		0.26	0.44
	Intergral (%)	5		14		8	73
	Total (%)	5		14		81	
*Oa*PAC WT 7FW	Chemical shift (ppm)	-130.5	-131.2	-132.9	-133.2	-134.2	
	Line width (ppm)	0.40	0.46	0.65	0.23	0.18	
	Intergral (%)	28	7	16	45	4	
	Total (%)	35		61		4	
*Sy*PixD	Chemical shift (ppm)	-121.6				-124.1	-123.8

Y8F 5FW	Line width (ppm)	0.28			0.20	0.25
	Intergral (%)	90			3	7
	Total (%)	90			10	
	Chemical shift (ppm)	-120.7	-123.1	-123.4	-124.1	-123.7
*Sy*PixD WT 5FW	Line width (ppm)	0.26	0.38	0.35	0.24	0.29
	Intergral (%)	12	24	14	9	41
	Total (%)	12	38		50	
	Chemical shift (ppm)	-130.6	-132.5		-134.2	-133.9
*Sy*PixD WT 7FW	Line width (ppm)	0.41	0.54		0.62	0.34
	Intergral (%)	23	29		12	36
	Total (%)	23	29		48	

Supplementary Table 2. Summary of AppA WT 5FW NMR spectrum deconvolution in 10%, 50%, and 90% D_2O/H_2O buffer condition (Supplementary Fig. 3). The Chemical shifts for the free 5FW and an unknown resonance experience a shift upon increasing the D_2O ratio.[1]

^{19}F	AppA WT			free 5FW			impurity		
	10%	50%	90%	10%	50%	90%	10%	50%	90%
Chemical shift (ppm)	-122.0	-122.0	-122.0	-124.8	-124.9	-125.0	-119.6	-120.7	-121.9
Line width (ppm)	0.20	0.24	0.24						

Supplementary Table 3. Parameters obtained from the kinetic analysis of AppA Y21F, *Oa*PAC Y6F, and *Sy*PixD Y8F in both H_2O and D_2O conditions. τ is the lifetime in ps, and β is the stretched parameter. The average lifetime $\langle\tau\rangle$ is calculated using the equation $\langle\tau\rangle = \frac{\tau}{\beta}\Gamma\left(\frac{1}{\beta}\right)$ where the gamma function is $\Gamma\left(\frac{1}{\beta}\right) = \int_0^{+\infty} t^{\beta^{-1}-1}\, e^{-t} dt$ $\left(\frac{1}{\beta} > 0\right)$. KIE (kinetic isotope effect) value is calculated using $k_{\mathrm{H}}/k_{\mathrm{D}} = \langle\tau\rangle_{\mathrm{D}}/\langle\tau\rangle_{\mathrm{H}}$.

		H_2O			D_2O			
	Parameters	τ (ps)	β	$<\tau>$ (ps)	τ (ps)	β	$<\tau>$ (ps)	KIE
AppA Y21F	fET	97	0.64	135	117	0.63	166	1.2
	fPT1	448	0.90	471	1049	1.00	1049	2.2
	rPT1	132	0.50	264	569	0.87	610	2.3
	rET	36	0.90	38	54	0.90	57	1.5
*Sy*PixD Y8F	fET	15	0.56	25	15	0.51	29	1.2
	fPT1	48	0.80	54	155	0.80	176	3.2
	fPT2	537	0.80	608	1141	0.80	1293	2.1
	rPT2	2741	0.80	3106	3494	0.80	3959	1.1
	rPT1	166	0.80	188	407	0.80	461	2.5
	rET	8.7	0.70	11	12	0.70	15	1.4
*Oa*PAC Y6F	fET	30	0.60	45	40	0.60	60	1.3
	fPT	140	0.70	177	194	0.70	246	1.4
	rPT	1964	0.70	2486	3697	0.70	4680	1.9
	rET	30	0.70	38	30	0.70	38	1.0

Supplementary Table 4. Multi-exponential fitting results of 760-nm single wavelength kinetics in the H_2O buffer (Fig. 6) using $y(t) = \sum A_i e^{-t/\tau} + A_{\text{offset}}$, of AppA W104F, *Oa*PAC W90F, and *Sy*PixD W91F, respectively.

		A_1 (%)	τ_1 (ps)	A_2 (%)	τ_2 (ps)	A_3 (%)	τ_3 (ps)	A_{offset}	$<\tau>$ (ps)
AppA	W104F	0.0	1.9	20.0	260	66.0	1338	14.0	935
*Oa*PAC	W90F	16.7	3.7	39.2	74	41.2	471	2.9	224
*Sy*PixD	W91F	28.3	1.8	38.0	30	29.2	317	4.5	105

Supplementary Table 5. Stretched exponential function fitting results of 760-nm single wavelength kinetics in the H_2O buffer (Fig. 6) using the stretched function $f(t) = Ae^{-(t/\tau)^{\beta}}$ and the parallel kinetic model considering the intersystem crossing pathway, of the dark-state AppA W104F, *Oa*PAC W90F, and *Sy*PixD W91F, respectively.

		τ_{fPCET} (ps)	β_{fPCET} (ps)	$<\tau>$ (ps)	τ_{ISC} (ps)
AppA	W104F	2431	0.71	3036	2200
*Oa*PAC	W90F	172	0.50	344	2800
*Sy*PixD	W91F	40	0.35	201	2600

Supplementary Table 6. The primers for site-directed mutagenesis.

Mutation	Primer Sequences
AppA	5'-CAGGGCGTCTTCTTCCAGTTCCTCGAAGGCCGCCCCGCC-3'
W64F	5'-GGCGGGGCGGCCTTCGAGGAACTGGAAGAAGACGCCCTG-3'
AppA	5'-GATCTGGTTTCCTGCTGCTTCCGCAGCCTG-3'
Y21F	5'-CAGGCTGCGGAAGCAGCAGGAAACCAGATC-3'
*Oa*PAC	5'-GGCAGCCATATGAAAAGACTCACCTTTATTAGTAAATTTTCCCGC-3'
Y6F	5'-GCGGGAAAATTTACTAATAAAGGTGAGTCTTTTCATATGGCTGCC-3'
*Sy*PixD	5'-GTACCGTTTGATTTTCAGCAGTCAGGGCATTCCC-3'
Y8F	5'-GGGAATGCCCTGACTGCTGAAAATCAAACGGTAC-3'
AppA	5'-GCCAAGCGCCGCTTTGCGGGATTCCACATGCAGCTCTCCTGCTCG-3'
W104F	5'-CGAGCAGGAGAGCTGCATGTGGAATCCCGCAAAGCGGCGCTTGGC-3'
*Oa*PAC	5'-GTCCAAGAAAGAATGTTTCCTGATTTCTCCATGCAAACGATTAACCTGG-3'
W90F	5'-CCAGGTTAATCGTTTGCATGGAGAAATCAGGAAACATTCTTTCTTGGAC-3'
*Sy*PixD	5'-GAAGAAACTTCGAGGTTTTCTCTATGCAAGCGATCACGGTG-3'
W91F	5'-CACCGTGATCGCTTGCATAGAGAAAACCTCGAAGTTTCTTC-3'

Supplementary Table 7. The amino acid sequences used for fluorotryptophan incorporation. The positions for labeling are indicated by the red-highlighted W.

Species	Amino Acid Sequences
$AppA_{1\text{-}124}$ W64F	MQHDLEADVTMTGSDLVSCCYRSLAAPDLTLRDLLDIVETSQAHNARAQLTGALFYS QGVFFQFLEGRPAAVAEVMTHIQRDRRHSNVEILAEEPIAKRRFAGWHMQLSCSEAD MRSLGLAESR

AppA$_{1-124}$ Y21F/W64F	MQHDLEADVTMTGSDLVSCCFRSLAAPDLTLRDLLDIVETSQAHNARAQLTGALFYS QGVFFQFLEGRPAAVAEVMTHIQRDRRHSNVEILAEEPIAKRRFAGWHMQLSCSEAD MRSLGLAESR
*Oa*PAC$_{1-102}$ WT	MKRLTYISKFSRPLSGDEIEAIGRISSQKNQQANVTGVLLCLDGIFFQILEGEAEKIDRIYE RILADERHTDILCLKSEVEVQERMFPDWSMQTINLDENTD
*Oa*PAC$_{1-102}$ Y6F	MKRLTFISKFSRPLSGDEIEAIGRISSQKNQQANVTGVLLCLDGIFFQILEGEAEKIDRIYE RILADERHTDILCLKSEVEVQERMFPDWSMQTINLDENTD
*Sy*PixD$_{1-150}$ WT	MSLYRLIYSSQGIPNLQPQDLKDILESSQRNNPANGITGLLCYSKPAFLQVLEGECEQVN ETYHRIVQDERHHSPQIIECMPIRRRNFEVWSMQAITVNDLSTEQVKTLVLKYSGFTTLR PSAMDPEQCLNFLLDIAKIYELSDNFFLDL
*Sy*PixD$_{1-150}$ Y8F	MSLYRLIFSSQGIPNLQPQDLKDILESSQRNNPANGITGLLCYSKPAFLQVLEGECEQVN ETYHRIVQDERHHSPQIIECMPIRRRNFEVWSMQAITVNDLSTEQVKTLVLKYSGFTTLR PSAMDPEQCLNFLLDIAKIYELSDNFFLDL

Supplementary Notes

Abbreviations

- Blue light using flavin (BLUF) domain
- Electron transfer (ET)
- Proton transfer (PT)
- Proton-coupled electron transfer (PCET)
- Kinetic isotope effect (KIE)
- Ground-state bleaching (GSB)
- Simulated emission (SE)
- Wild type (WT)
- Flavin mononucleotide (FMN)
- Tyrosine (Tyr / Y)
- Glutamine (Gln / Q)
- Tryptophan (Trp / W)
- Phenylalanine (Phe / F)
- Concerted electron–proton transfer (CEPT)
- Proton relay (PR) intermediate
- Charge separated (CS) intermediate
- Intersystem crossing (ISC)
- Evolution-Associated Difference Spectra (EADS)
- Species-Associated Difference Spectra (SADS)
- Kinetic isotope effects (KIE)
- Transient absorption (TA)
- Di-radical (DR) pair
- Nuclear magnetic resonance spectroscopy (NMR)
- 4-fluorotryptophan (4FW)
- 5-fluorotryptophan (5FW)
- 6-fluorotryptophan (6FW)
- 7-fluorotryptophan (7FW)

Supplementary Methods

Evolution Associated Difference Spectra (EADS) Analysis.

Transient absorption 2D contour maps (Fig. 3a-c) in H_2O and D_2O conditions were first analyzed with sequential model fitting then the corresponding EADS were obtained. But the model used in EADS analysis is sequential, so it is unable to represent the actual physical process in most cases. Usually, EADS analysis was conducted on the raw TA data (Fig. 3a-c, upper panel), as well as the data where FMN^* contribution was subtracted (Fig. 3a-c, lower panel).

Subtract FMN^* contribution from the W_{out} contribution removed matrix.

The W_{out} contribution has been removed from raw TA data based on Supplementary Fig. 9. The FMN^* subtraction method, previously developed by Tahara and our group, was applied. The FMN^* matrix was reproduced using Equation [S1], where $[\mathrm{Kin1}]_{1\times n}$ represents the FMN^* decay kinetics, $[\mathrm{Spectrum1}]_{m\times 1}$ represents the pure FMN^* spectrum, and A1 is the weight of the spectra and kinetic vector. The kinetics at 760 nm, where FMN^* absorbs predominantly, were used as $[\mathrm{Kin1}]_{1\times n}$, while the first EADS was assumed to be the pure spectrum of FMN^* when the other species had yet to be involved. A1 was determined by normalizing the first EADS at 760 nm. As a result, the $[\mathrm{FMN}^*]_{m\times n}$ matrix was subtracted from the matrix where the W_{out} contribution was pre-removed using the YnF/WnF TA matrix. FMN^* subtraction is a critical step in data analysis as it reveals the intermediate species which is otherwise overwhelmed by FMN^* signals and therefore not identified.

$$[\mathrm{FMN}^*]_{m\times n} = \mathrm{A1}\cdot[\mathrm{Spectrum1}]_{m\times 1}\cdot[\mathrm{Kin1}]_{1\times n} \qquad [\mathrm{S1}]$$

The general target analysis setting.

Based on the results from EADS analysis and FMN^* subtraction analysis, a physically-relevant model was constructed for target analysis. In the target analysis, we have invoked the stretched exponential function $f(t) = Ae^{-(t/\tau)^{\beta}}$ in the global fitting, which has been used to describe kinetic heterogeneity when the physical process is coupled with the solvation on the similar time scale.[5-6] For a PT-involved process, the dynamic heterogeneity may also arise from H-bond fluctuations as the rate of PT is very sensitive to the H-bond distances and angles. In the function, τ is the lifetime, t is the time delay, and A is the amplitude. Thus, the $\frac{1}{\tau_i}$ was replaced with $\beta_i\frac{t^{\beta_i-1}}{\tau_i{}^{\beta_i}}$ in all of the differential rate equations in this study, where i indicates the respective process. Equation [S2] is used to calculate the averaged lifetime from the stretch function where the gamma function (Γ) is shown in Equation [S3]. The lifetimes given in Fig. 4, Fig. 6 (main text) and Supplementary Fig. 8 are averaged lifetimes. In this study, the instrument response function (IRF) of ~100 fs was convoluted with the kinetics from the differential rate equations.

At the zeroth time delay, the total concentration of the excited species (FMN*) was assumed to be n_0 during the target analysis.

$$\langle\tau\rangle = \frac{\tau}{\beta}\Gamma(\frac{1}{\beta}) \quad [S2]$$

$$\Gamma\left(\frac{1}{\beta}\right) = \int_0^{+\infty} t^{\beta^{-1}-1} e^{-t}\mathrm{d}t \; (\frac{1}{\beta} > 0) \quad [S3]$$

Target analysis – single-rocking model for AppA Y21F.

As described in the main test, in AppA Y21F, FMN* undergoes fET (Step 1, scheme in Supplementary Fig. 6B) to form $FMN^{\cdot-}/WH^{\cdot+}$ (CS state) followed by fPT1 (Step 2) to form $FMN^{\cdot-}/W^{\cdot}$ (PR intermediate), then $FMN^{\cdot-}/W^{\cdot}$ undergoes rPT1 (Step 3) to regenerate $FMN^{\cdot-}/WH^{\cdot+}$. $FMN^{\cdot-}$ and $WH^{\cdot+}$ then recombine to ground state (rET, Step 6). To obtain the parameters (*i.e.*, τ_{fET}, β_{fET}, τ_{fPT1}, β_{fPT1}, τ_{rPT1}, β_{rPT1}, τ_{rET}, and β_{rET}), 760 nm, 750 nm, 621 nm, 550 nm, 517 nm, 501 nm, 445 nm, 377 nm and 357 nm were selected to fit simultaneously globally with equations [S4-S6]. In this fitting, 621 nm, 517 nm and 501 nm were fitted with equations [S5-S6]. 760 nm, 750 nm, 550 nm, 445 nm, 377 nm and 357 nm were fitted with Equations [S4-S6].

$$\frac{d}{dt}[\mathrm{FMN}^*(t)] = -\frac{1}{\tau_{\mathrm{fET}}}[\mathrm{FMN}^*(t)] \quad [S4]$$

$$\frac{d}{dt}[\mathrm{FMN}^{\cdot-}/\mathrm{WH}^{\cdot+}(t)] = \frac{1}{\tau_{\mathrm{fET}}}[\mathrm{FMN}^*(t)] - \frac{1}{\tau_{\mathrm{fPT1}}}[\mathrm{FMN}^{\cdot-}/\mathrm{WH}^{\cdot+}(t)] - \frac{1}{\tau_{\mathrm{rET}}}[\mathrm{FMN}^{\cdot-}/\mathrm{WH}^{\cdot+}(t)] + \frac{1}{\tau_{\mathrm{rPT1}}}[\mathrm{FMN}^{\cdot-}/\mathrm{W}^{\cdot}(t)] \quad [S5]$$

$$\frac{d}{dt}[\mathrm{FMN}^{\cdot-}/\mathrm{W}^{\cdot}(t)] = \frac{1}{\tau_{\mathrm{fPT1}}}[\mathrm{FMN}^{\cdot-}/\mathrm{WH}^{\cdot+}(t)] - \frac{1}{\tau_{\mathrm{rPT1}}}[\mathrm{FMN}^{\cdot-}/\mathrm{W}^{\cdot}(t)] \quad [S6]$$

By using the parameters as listed in Supplementary Table 3 (τ_{fET}, β_{fET}, τ_{fPT1}, β_{fPT1}, τ_{rPT1}, β_{rPT1}, τ_{rET}, and β_{rET}), the Species-associated differential spectra (SADS) and the corresponding kinetic traces of the species can be deconvoluted by using Equations [S7-S10] to analyze the data. $[\mathrm{Kin(species)}]_{1\times n}$ are the kinetics of the species and $\mathrm{A(species)}\cdot[\mathrm{SADS(species)}]_{m\times 1}$ are the SADS, whereas the TA matrix is the sum of all species-associated matrixes as indicates in Equation [S10].

$$[\mathrm{FMN}^*]_{m\times n} = \mathrm{A}(\mathrm{FMN}^*)\cdot[\mathrm{SADS}(\mathrm{FMN}^*)]_{m\times 1}\cdot[\mathrm{Kin}(\mathrm{FMN}^*)]_{1\times n} \quad [S7]$$

$$[\mathrm{FMN}^{\cdot-}/\mathrm{WH}^{\cdot+}]_{m\times n} = \mathrm{A}(\mathrm{FMN}^{\cdot-}/\mathrm{WH}^{\cdot+})\cdot[\mathrm{SADS}(\mathrm{FMN}^{\cdot-}/\mathrm{WH}^{\cdot+})]_{m\times 1}\cdot[\mathrm{Kin}(\mathrm{FMN}^{\cdot-}/\mathrm{WH}^{\cdot+})]_{1\times n} \quad [S8]$$

$$[\mathrm{FMN}^{\cdot-}/\mathrm{W}^{\cdot}]_{m\times n} = \mathrm{A}(\mathrm{FMN}^{\cdot-}/\mathrm{W}^{\cdot})\cdot[\mathrm{SADS}(\mathrm{FMN}^{\cdot-}/\mathrm{W}^{\cdot})]_{m\times 1}\cdot[\mathrm{Kin}(\mathrm{FMN}^{\cdot-}/\mathrm{W}^{\cdot})]_{1\times n} \quad [S9]$$

$$[\mathrm{TA\ Data}]_{m\times n} = [\mathrm{FMN}^*]_{m\times n} + [\mathrm{FMN}^{\cdot-}/\mathrm{WH}^{\cdot+}]_{m\times n} + [\mathrm{FMN}^{\cdot-}/\mathrm{W}^{\cdot}]_{m\times n} \quad [S10]$$

Target analysis – single-rocking model for *Oa*PAC Y6F.

As described in the main text, in *Oa*PAC Y6F, FMN* undergoes fET (Step 1, scheme in Supplementary Fig. 6A) to form $FMN^{\cdot-}/WH^{\cdot+}$ (CS state) followed by fPT1+fPT2 (Step 2 and Step 4) to form $FMNH^{\cdot}/W^{\cdot}$ (DR

intermediate), then $FMNH^{\cdot}/W^{\cdot}$ undergo rPT2+1 (Step 5 and Step 3) to regenerate $FMN^{\cdot-}/WH^{\cdot+}$. $FMN^{\cdot-}$ and $WH^{\cdot+}$ then recombine to ground state (rET, Step 6). To obtain the parameters (*i.e.*, τ_{fET}, β_{fET}, τ_{fPT}, β_{fPT}, τ_{rPT}, β_{rPT}, τ_{rET}, and β_{rET}), 760 nm, 750 nm, 665 nm, 621 nm, 550 nm, 517 nm, 501 nm, 445 nm and 357 nm were selected to fit simultaneously globally. In this fitting, 760 nm, 750 nm, 665 nm and 357 nm were fitted with Equations [S11-S12]. 621 nm, 517 nm and 501 nm were fitted with Equations [S12-S13]. 550 nm and 445 nm were fitted with Equations [S11-S13].

$$\frac{d}{dt}[\mathrm{FMN}^*(t)] = -\frac{1}{\tau_{\mathrm{fET}}}[\mathrm{FMN}^*(t)] \quad \text{[S11]}$$

$$\frac{d}{dt}[\mathrm{FMN}^{\cdot-}/\mathrm{WH}^{\cdot+}(t)] = \frac{1}{\tau_{\mathrm{fET}}}[\mathrm{FMN}^*(t)] - \frac{1}{\tau_{\mathrm{fPT}}}[\mathrm{FMN}^{\cdot-}/\mathrm{WH}^{\cdot+}(t)] - \frac{1}{\tau_{\mathrm{rET}}}[\mathrm{FMN}^{\cdot-}/\mathrm{WH}^{\cdot+}(t)] + \frac{1}{\tau_{\mathrm{rPT}}}[\mathrm{FMNH}^{\cdot}/\mathrm{W}^{\cdot}(t)] \quad \text{[S12]}$$

$$\frac{d}{dt}[\mathrm{FMNH}^{\cdot}/\mathrm{W}^{\cdot}(t)] = \frac{1}{\tau_{\mathrm{fPT}}}[\mathrm{FMN}^{\cdot-}/\mathrm{WH}^{\cdot+}(t)] - \frac{1}{\tau_{\mathrm{rPT}}}[\mathrm{FMNH}^{\cdot}/\mathrm{W}^{\cdot}(t)] \quad \text{[S13]}$$

By using the parameters as listed in Supplementary Table 3 (τ_{fET}, β_{fET}, τ_{fPT}, β_{fPT}, τ_{rPT}, β_{rPT}, τ_{rET}, and β_{rET}), the SADS and the corresponding kinetic traces of the species can be deconvoluted by using Equations [S14-S17] to analyze the data. $[\mathrm{Kin(species)}]_{1\times n}$ are the kinetics of the species and $\mathrm{A(species)} \cdot [\mathrm{SADS(species)}]_{m\times 1}$ are the SADS, whereas the TA matrix is the sum of all species-associated matrixes as indicates in Equation [S17].

$$[\mathrm{FMN}^*]_{m\times n} = \mathrm{A}(\mathrm{FMN}^*) \cdot [\mathrm{SADS}(\mathrm{FMN}^*)]_{m\times 1} \cdot [\mathrm{Kin}(\mathrm{FMN}^*)]_{1\times n} \quad \text{[S14]}$$

$$[\mathrm{FMN}^{\cdot-}/\mathrm{WH}^{\cdot+}]_{m\times n} = \mathrm{A}(\mathrm{FMN}^{\cdot-}/\mathrm{WH}^{\cdot+}) \cdot [\mathrm{SADS}(\mathrm{FMN}^{\cdot-}/\mathrm{WH}^{\cdot+})]_{m\times 1} \cdot [\mathrm{Kin}(\mathrm{FMN}^{\cdot-}/\mathrm{WH}^{\cdot+})]_{1\times n} \quad \text{[S15]}$$

$$[\mathrm{FMNH}^{\cdot}/\mathrm{W}^{\cdot}]_{m\times n} = \mathrm{A}(\mathrm{FMNH}^{\cdot}/\mathrm{W}^{\cdot}) \cdot [\mathrm{SADS}(\mathrm{FMNH}^{\cdot}/\mathrm{W}^{\cdot})]_{m\times 1} \cdot [\mathrm{Kin}(\mathrm{FMNH}^{\cdot}/\mathrm{W}^{\cdot})]_{1\times n} \quad \text{[S16]}$$

$$[\mathrm{TA\ Data}]_{m\times n} = [\mathrm{FMN}^*]_{m\times n} + [\mathrm{FMN}^{\cdot-}/\mathrm{WH}^{\cdot+}]_{m\times n} + [\mathrm{FMNH}^{\cdot}/\mathrm{W}^{\cdot}]_{m\times n} \quad \text{[S17]}$$

Target analysis – double-rocking model for *Sy*PixD Y8F.

As described in the main test, in *Sy*PixD Y8F mutant, FMN* proceeds with fET (Step 1, scheme in Supplementary Fig. 6A) to form $FMN^{\cdot-}/WH^{\cdot+}$ (CS state) followed by fPT1 (Step 2) to generate $FMN^{\cdot-}/W^{\cdot}$ (PR intermediate). The PR intermediate then undergoes fPT2 (Step 4) to generate $FMNH^{\cdot}/W^{\cdot}$ (DR intermediate). $FMNH^{\cdot}/W^{\cdot}$ decays back to ground state via sequential rPT2/rPT1/rET (Step 5/Step 3/Step 6). Kinetics from nine wavelengths (760 nm, 750 nm, 665 nm, 621 nm, 550 nm, 517 nm, 501 nm, 445 nm and 357 nm) were selected to fit the data simultaneously in order to obtain the parameters (*i.e.*, τ_{fET}, β_{fET}, τ_{fPT1}, β_{fPT1}, τ_{fPT2}, β_{fPT2}, τ_{rPT2}, β_{rPT2}, τ_{rPT1}, β_{rPT1}, τ_{rET}, and β_{rET}) to describe the double-rocking model. 760 nm, 750 nm, 665 nm and 357 nm were fitted with Equations [S18-S20]. 621 nm, 517 nm, 501 nm were fitted with Equations [S19-S21]. 550 nm and 445 nm were fitted with Equations [S18-S21]. The obtained parameters are listed in Supplementary Table 3.

$$\frac{d}{dt}[\mathrm{FMN}^*(t)] = -\frac{1}{\tau_{\mathrm{fET}}}[\mathrm{FMN}^*(t)] \quad \text{[S18]}$$

$$\frac{d}{dt}[\mathrm{FMN}^{\cdot-}/\mathrm{WH}^{\cdot+}(t)] = \frac{1}{\tau_{\mathrm{fET}}}[\mathrm{FMN}^{*}(t)] - \frac{1}{\tau_{\mathrm{fPT1}}}[\mathrm{FMN}^{\cdot-}/\mathrm{WH}^{\cdot+}(t)]$$

$$-\frac{1}{\tau_{\mathrm{rET}}}[\mathrm{FMN}^{\cdot-}/\mathrm{WH}^{\cdot+}(t)] + \frac{1}{\tau_{\mathrm{rPT1}}}[\mathrm{FMN}^{\cdot-}/\mathrm{W}^{\cdot}(t)] \quad [S19]$$

$$\frac{d}{dt}[\mathrm{FMN}^{\cdot-}/\mathrm{W}^{\cdot}(t)] = \frac{1}{\tau_{\mathrm{fPT1}}}[\mathrm{FMN}^{\cdot-}/\mathrm{WH}^{\cdot+}(t)] - \frac{1}{\tau_{\mathrm{rPT1}}}[\mathrm{FMN}^{\cdot-}/\mathrm{W}^{\cdot}(t)]$$

$$-\frac{1}{\tau_{\mathrm{fPT2}}}[\mathrm{FMN}^{\cdot-}/\mathrm{W}^{\cdot}(t)] + \frac{1}{\tau_{\mathrm{rPT2}}}[\mathrm{FMNH}^{\cdot}/\mathrm{W}^{\cdot}(t)] \quad [S20]$$

$$\frac{d}{dt}[\mathrm{FMNH}^{\cdot}/\mathrm{W}^{\cdot}(t)] = \frac{1}{\tau_{\mathrm{fPT2}}}[\mathrm{FMN}^{\cdot-}/\mathrm{W}^{\cdot}(t)] - \frac{1}{\tau_{\mathrm{rPT2}}}[\mathrm{FMNH}^{\cdot}/\mathrm{W}^{\cdot}(t)] \quad [S21]$$

By using the parameters obtained from above, Equations [S22-S26] can be used to deconvoluted the SADS and the corresponding kinetic traces of the species to analyze the data. The TA matrix is the sum of all species-associated matrixes as indicates in Equation [S26].

$$[\mathrm{FMN}^{*}]_{m\times n} = \mathrm{A}(\mathrm{FMN}^{*}) \cdot [\mathrm{SADS}(\mathrm{FMN}^{*})]_{m\times 1} \cdot [\mathrm{Kin}(\mathrm{FMN}^{*})]_{1\times n} \quad [S22]$$

$$[\mathrm{FMN}^{\cdot-}/\mathrm{WH}^{\cdot+}]_{m\times n} = \mathrm{A}(\mathrm{FMN}^{\cdot-}/\mathrm{WH}^{\cdot+}) \cdot [\mathrm{SADS}(\mathrm{FMN}^{\cdot-}/\mathrm{WH}^{\cdot+})]_{m\times 1} \cdot [\mathrm{Kin}(\mathrm{FMN}^{\cdot-}/\mathrm{WH}^{\cdot+}]_{1\times n} \quad [S23]$$

$$[\mathrm{FMN}^{\cdot-}/\mathrm{W}^{\cdot}]_{m\times n} = \mathrm{A}(\mathrm{FMN}^{\cdot-}/\mathrm{W}^{\cdot}) \cdot [\mathrm{SADS}(\mathrm{FMN}^{\cdot-}/\mathrm{W}^{\cdot})]_{m\times 1} \cdot [\mathrm{Kin}(\mathrm{FMN}^{\cdot-}/\mathrm{W}^{\cdot}]_{1\times n} \quad [S24]$$

$$[\mathrm{FMNH}^{\cdot}/\mathrm{W}^{\cdot}]_{m\times n} = \mathrm{A}(\mathrm{FMNH}^{\cdot}/\mathrm{W}^{\cdot}) \cdot [\mathrm{SADS}(\mathrm{FMNH}^{\cdot}/\mathrm{W}^{\cdot})]_{m\times 1} \cdot [\mathrm{Kin}(\mathrm{FMNH}^{\cdot}/\mathrm{W}^{\cdot})]_{1\times n} \quad [S25]$$

$$[\mathrm{TA\ Data}]_{m\times n} = [\mathrm{FMN}^{*}]_{m\times n} + [\mathrm{FMN}^{\cdot-}/\mathrm{WH}^{\cdot+}]_{m\times n} + [\mathrm{FMN}^{\cdot-}/\mathrm{W}^{\cdot}]_{m\times n} + [\mathrm{FMNH}^{\cdot}/\mathrm{W}^{\cdot}]_{m\times n} \quad [S26]$$

The criteria for determining the goodness of matrix deconvolution.

In the process of matrix deconvolution, further fitting refinements are often necessary to improve the accuracy of the analysis. These refinements involve adjusting the initial parameter values obtained from the initial deconvolution and applying constraints during the multiple-wavelength global fitting. These refinements aim to fine-tune the parameters and obtain the best possible set of parameters that accurately represent the system dynamics.

There are several criteria to determine if a target analysis is well performed or not. First, the SADS spectra agreement between H_2O and D_2O: The electronic absorption properties should not be significantly affected by the exchange of hydrogen (H) with deuterium (D). Therefore, the SADS spectra obtained in H_2O and D_2O conditions should show similarity, indicating that the deconvolution accurately captures the spectral features. Second, a small residual matrix: The residual matrix, representing the difference between the experimental data and the fitted data, should be small. A small residual matrix indicates a good fit between the observed data and the model used in the deconvolution. Third, the goodness of the fit of important kinetics at representative wavelengths: The target analysis should provide a good fit for the representative wavelengths. Fourth, consistency of SADS with proposed intermediates: The SADS obtained from the deconvolution should be consistent with the proposed intermediates involved in the system. The intermediates' characteristic absorption features observed in the SADS should align

with the expected behavior based on prior knowledge or theoretical predictions. This consistency provides reasonableness and confidence in the accuracy of the deconvolution process.

Supplementary References

1. Gee, C. T. *et al.* Protein-observed ^{19}F-NMR for fragment screening, affinity quantification and druggability assessment. *Nat. Protoc.* **11**, 1414–1427 (2016).
2. Lacombat, F. *et al.* Ultrafast Oxidation of a Tyrosine by Proton-Coupled Electron Transfer Promotes Light Activation of an Animal-like Cryptochrome. *J. Am. Chem. Soc.* **141**, 13394–13409 (2019).
3. Kang, X.-W. *et al.* Direct Observation of Ultrafast Proton Rocking in the BLUF Domain. *Angew. Chemie - Int. Ed.* **61**, e202114423 (2022).
4. Geoghegan, K. F. *et al.* Spontaneous *α*-*N*-6-phosphogluconoylation of a 'His tag' in *Escherichia coli*: The cause of extra mass of 258 or 178 Da in fusion proteins. *Anal. Biochem.* **267**, 169–184 (1999).
5. Li, J. *et al.* Dynamics and mechanism of repair of ultraviolet-induced (6-4) photoproduct by photolyase. *Nature* **466**, 887–890 (2010).
6. Zhang, M., Wang, L. & Zhong, D. Photolyase: Dynamics and electron-transfer mechanisms of DNA repair. *Arch. Biochem. Biophys.* **632**, 158–174 (2017).
